# Resistance Training Practices of Brazilian Olympic Sprint and Jump Coaches: Toward a Deeper Understanding of Their Choices and Insights (Part III)

**DOI:** 10.5114/jhk/182888

**Published:** 2024-02-02

**Authors:** Irineu Loturco, Santiago Zabaloy, Lucas A. Pereira, Túlio B. M. A. Moura, Valter P. Mercer, Fernandes Victor, Adam Zając, Aleksander Matusinski, Tomás T. Freitas, Chris Bishop

**Affiliations:** 1 NAR—Nucleus of High Performance in Sport, São Paulo, Brazil.; 2 Department of Human Movement Sciences, Federal University of São Paulo, São Paulo, Brazil.; 3 Department of Sport, Health, and Exercise Science, University of South Wales, Pontypridd, Wales, United Kingdom.; 4 Faculty of Physical Activity and Sports, University of Flores, Buenos Aires, Argentina.; 5 Institute of Sport Sciences, The Jerzy Kukuczka Academy of Physical Education in Katowice, Katowice, Poland.; 6 Department of Exercise and Sport Performance, The Jerzy Kukuczka Academy of Physical Education in Katowice, Katowice, Poland.; 7 UCAM Research Center for High Performance Sport, UCAM Universidad Católica de Murcia, Murcia, Spain.; 8 Facultad de Deporte, UCAM Universidad Católica de Murcia, Murcia, Spain.; 9 London Sport Institute, Middlesex University, London, United Kingdom.

**Keywords:** athletic performance, track and field, sprint speed, muscle strength, strength training, athletes

## Abstract

In the final part of this three-article collection on the training strategies of Brazilian Olympic sprint and jump coaches, we provide a detailed description of the resistance training methods and exercises most commonly employed by these speed experts. Always with the objective of maximizing the sprint and jump capabilities of their athletes, these experienced coaches primarily utilize variable, eccentric, concentric, machine-based, isometric, complex, and isoinertial resistance training methods in their daily practices. Squats (in their different forms), Olympic weightlifting, ballistics, hip thrusts, lunges, calf raises, core exercises, leg curls, stiff-leg deadlifts, and leg extension are the most commonly prescribed exercises in their training programs, during both the preparatory and competitive periods. Therefore, the current manuscript comprehensively describes and examines these methods, with the additional aim of extrapolating their application to other sports, especially those where sprint speed is a key performance factor.

## Introduction

The improvement of strength and power qualities stands as a fundamental priority across a wide variety of sports (Suchomel et al., 2016, 2018). Indeed, it is well-established that muscle strength and power provide a solid foundation for the proper development of various other physical abilities, including linear sprinting and change of direction (COD) speed, jumping, and even endurance-related capacities (e.g., running economy) (Suchomel et al., 2016, 2018). Resistance training, in all its different forms and types (e.g., eccentric versus concentric; ballistic versus non-ballistic), is, unquestionably, the most popular and effective method to enhance strength and power adaptations in athletes from multiple sports, levels, and age-categories (Cormier et al., 2021; Dowse et al., 2020; Haff and Nimphius, 2012; Suchomel et al., 2021). Therefore, it is expected that track and field coaches will consider resistance training methods, and their broad range of strategies (e.g., complex training, ballistic exercises), one of the more relevant and crucial parts of their training planning and programs (Deweese et al., 2015; Healy et al., 2021; Suchomel et al., 2018). This is particularly evident in sprint and jump events, athletic disciplines in which athletes are distinguished by their exceptional levels of relative strength and power (Loturco et al., 2021a, 2021c; Young et al., 1995).

Previous studies involving sprint and jump coaches have already addressed these issues, elucidating the main goals and primary objectives for implementing and using diverse strength-power training regimens and exercises, while also outlining the purposes and objectives behind this rationale. In a survey on the practices of sprinting coaches, Healy et al. (2021) revealed that sprint coaches selected resistance exercises based on three principal reasons: (1) performance adaptations; (2) practicality; and 3) targeting muscles/muscle groups that mimic sprinting technique. Approximately 71% of these coaches (41 sprint coaches working in Ireland with coaching experience of ~8.4 ± 6.4 years, including 43.9, 36.6, and 19.5% of coaches who worked with international, national, and regional level athletes, respectively) reported that, in their views, resistance training was “very important” for sprinters. In a practical study regarding “Strength and Conditioning Considerations for the 100-m Sprinter”, Cissik (2010) reinforces these points, as well as highlights some important questions that should be taken into account when designing resistance training programs for elite sprinters: the short-time duration of the race (~10 s); the explosive nature of the event; the necessity to constantly apply force against the ground; and, finally, the unilateral characteristic of the sprint (i.e., only one leg is in contact with the ground at a time).

In summary, and similarly to what occurs in multiple individual and team-sports, for sprint and jump coaches, unquestionably, similarity (with sport-specific techniques) and effectiveness are the main reasons for selecting and applying a range of strength-power exercises and resistance training methods (Healy et al., 2021; Loturco et al., 2023a, 2023b). These aims are also common among Brazilian Olympic sprint and jump coaches (Loturco et al., 2023b), who have mentioned that resistance training schemes are frequently utilized to increase neuromuscular readiness during the competitive period and to reduce perceptual fatigue, potentially minimizing the risk of injuries across successive competitions. Within a series of essential strategies reported by these coaches, we can highlight, for example, variable resistance training (VRT) as well as squats and their variations as the most frequently used methods and exercises, respectively (Loturco et al., 2023b) (Tables 1 and 2 show the complete list of methods and exercises most commonly used by these coaches, along with their relative frequency of utilization). In the current article—the final of a three-article special collection on Olympic sprint and jump coaching practices—we will list, describe, and critically analyze each of these strategies, with the aim of exploring the experience of these speed experts and transferring this knowledge to practitioners from various sports.

**Table 1:** Resistance training methods most commonly employed by Brazilian Olympic sprint and jump coaches.

Resistance training method	% of utilization
Variable resistance	79%
Eccentric	63%
Concentric	58%
Machine-based	53%
Isometric	37%
*Complex	21%
Isoinertial	16%

**Note:** *In the original study (Loturco et al., 2023b), complex training was identified as a method primarily employed for speed development. However, for the purpose of clarity and understanding, we categorized and described this method as a resistance training method.

**Table 2:** Resistance training exercises most frequently utilized by Brazilian Olympic sprint and jump coaches during both the preparatory and competitive periods.

Exercise	% of utilization
Squat and its variations	100%
Olympic weightlifting and derivatives	100%
Ballistic exercises	47%
Hip thrust	26%
Lunge	21%
Calf raises	15%
Core exercises	10%
Leg curl	5%
Leg extension	5%
Stiff-leg deadlift	5%

## Resistance Training Programming

**Figure 1:**
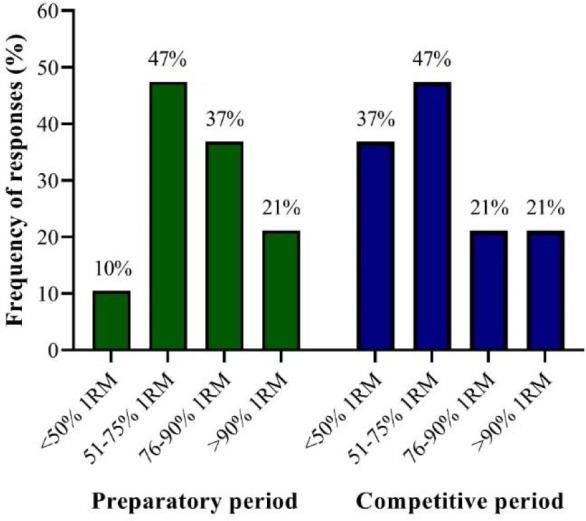
Frequency of responses regarding the relative percentage of resistance training loads (% 1RM) utilized by Brazilian Olympic sprint and jump coaches during both preparatory and competitive periods.

As fully detailed in the original manuscript (Loturco et al., 2023b) that underlies this three-article collection, the typical resistance training program prescribed by Olympic Brazilian sprint and jump coaches during the off-season period consists of 2–4 sessions per week. On average, each session lasts more than 60 min and comprises 3–4 sets of 4–6 or 10–12 repetitions per exercise. Throughout the competitive period, resistance training sessions are primarily conducted twice a week, usually lasting between 46 and 60 min. Each session, in general, includes 3–4 sets of 4–6 repetitions per exercise. Strength-power training loads are mainly determined by movement velocity (i.e., velocity-based training), although some coaches reveal using one-repetition maximum (1RM) testing or “athlete-dependent” methods (Figure 1 displays the relative percentage of resistance training loads [% 1RM] used by Brazilian Olympic sprint and jump coaches during both the preparatory and competitive periods). Most coaches stated that they implemented more adaptable and flexible training programs, tailoring the training content and loads to meet the individual demands, needs, and objectives of their athletes. Finally, the resistance training sessions were predominantly performed on the same day or within 24 h after the specific track and field training sessions. For further information regarding the programming practices of Brazilian Olympic sprint and jump coaches, please consult the manuscript (Loturco et al., 2023b) that originated this collection, also published in an open-access format in the *Journal of Human Kinetics*.

## Resistance Training Methods

The resistance training methods most frequently employed by Brazilian Olympic sprint and jump coaches in their daily practices are listed in Table 1(Loturco et al., 2023b). This table serves as a brief introduction to the comprehensive description and analysis of each method, as presented in this manuscript.

## Variable Resistance Training

Interestingly, VRT appears as the most commonly utilized resistance training method among Brazilian Olympic sprint and jump coaches (mentioned by ~80% of these coaches) (Loturco et al., 2023b). Although widely used in numerous sports (e.g., soccer, cricket, rugby, basketball, etc.) and countries (Loturco et al., 2022a; Weldon et al., 2021a, 2021c, 2022), in these sports, VRT is usually the third or fourth preferred resistance training method, as compared to more “traditional” training approaches, such as eccentric, concentric, and isometric training methods (Loturco et al., 2022a; Weldon et al., 2021a, 2021c, 2022). From a general perspective, the extensive use of VRT in elite sports may be related to three different factors: (1) low cost of implementation, ease of use, and portability—as VRT is predominantly performed with elastic bands; (2) the ability and efficiency to mimic sport-specific actions and transfer them to various sports; and (3) the mechanical advantages potentially associated with this training strategy (Loturco et al., 2022d, 2023b; Saeterbakken et al., 2014; Soria-Gila et al., 2015). Specifically for Brazilian sprint and jump coaches, the scarcity of adequate track and field training facilities and equipment across the country (i.e., Brazil) frequently requires coaches and practitioners to adapt and employ alternative training methods (which may explain the high usage of useful and practical elastic bands as training tools) (Loturco et al., 2023b). Together, these factors probably make VRT the most common resistance training method, not only within sprint and jump events, but also for other track and field disciplines in the Brazilian context (Loturco et al., 2023b).

In addition to these reasons, more recently, VRT has been proposed as an alternative and effective method for optimizing strength gains in resistance trained subjects (Loturco et al., 2022d; Soria-Gila et al., 2015). Overall, VRT is prescribed using a combination of elastic bands and free weights with the objective of maximizing force application in the final phase of the movement, when the elastic band is overstretched. It is important to note that the elastic properties of this material allow the initial portion of the concentric action, when the elastic band is not stretched, to be performed at faster velocities since the external resistance is relatively lower compared to the final portion (Kubo et al., 2018; Loturco et al., 2022d; Saeterbakken et al., 2016). A potential drawback of this variability in external resistance is the difficulty in quantifying the exact resistance provided by the elastic bands. An alternative way to establish a “target training zone” is by measuring the reductions in movement velocity caused by each band and comparing these values with those reported in previous velocity-based training studies. This can be easily achieved using a linear position or a velocity transducer, for example (Loturco et al., 2022d). In fact, Saeterbakken et al. (2014) observed gradual increases in the resistance values of 93%, 105%, and 117% in the lower, middle, and upper positions, respectively, during back squats executed with elastic bands compared to regular back squats (i.e., exercise with only free weights as loading) using a 6-repetition maximum load. This is indeed an exclusive and typical characteristic of VRT, which may lead to a series of potential benefits and increased responses, such as improvements in neuromuscular control and activation, especially in the latter stages of the lifting (Aboodarda et al., 2013; Andersen et al., 2015; Loturco et al., 2022d; Rivière et al., 2017). This aspect may be highly interesting for sprinters and jumpers who need to apply higher levels of force, for example, in the final phases of the ground contact during the step cycle (Hunter et al., 2005; Loturco et al., 2015a; Weyand et al., 2000; Young and Choice, 2007). Different studies have confirmed this “theoretical” superiority of VRT over traditional resistance training in terms of maximum strength development (Anderson et al., 2008; Loturco et al., 2022d) (with some studies reporting improvements ~2 and 3 times greater for the bench press and squat 1RM, respectively) (Anderson et al., 2008). An important point to highlight is that a longer study (i.e., ≥ 10 weeks) did not find significant differences between groups that trained under traditional or variable training conditions (Andersen et al., 2015). This suggests that advantages provided by VRT may be more evident during short-term interventions (i.e., 4–8 weeks) (Loturco et al., 2022d), which could be a key advantage within team-sport contexts, where pre-seasons (as well as off-seasons) are usually of very short duration (i.e., 4–6 weeks) (Loturco et al., 2022a, 2022d).

The implementation of combined training (VRT + free weight exercises) has also been shown to be effective in developing power-related qualities, provided that certain aspects are considered. In this regard, VRT should be prescribed in conjunction with light-loads (i.e., bar-velocities ranging from 1.0 to 1.2 m•s^−1^; ~30–40% half squat 1RM) (Loturco et al., 2023c) and preferably using ballistic exercises (e.g., loaded jump squats) (Loturco et al., 2022d). Nevertheless, it is worth noting that traditional movements (without using elastic bands) still tend to be superior for developing muscle power under ballistic conditions, which may be logical from some perspectives. Without reducing takeoff or throwing velocity in the final portion of the concentric phase during a ballistic action (the “natural effect” of using elastic bands), we can also increase power production and, potentially, improve the transfer to power performance (Cormie et al., 2011; Loturco et al., 2022d). Curiously, the same mechanical phenomenon (i.e., the gradual increase in force application across the entire range of motion) is likely responsible for the higher magnitude in neuromuscular adaptations—resulting in superior enhancements in maximum strength and jumping capacity—commonly observed after the implementation of VRT, in comparison with more traditional free weight training schemes (Anderson et al., 2008; Joy et al., 2016; Loturco et al., 2022d). Indeed, the progressive increase in the rate of elastic tension that occurs during VRT requires athletes to continuously accelerate the load throughout the range of motion (Anderson et al., 2008; Joy et al., 2016; Soria-Gila et al., 2015). This is in contrast to traditional free weight training, where athletes must decelerate during the “sticking period”—the phase of the movement characterized by a temporary and expected reduction in force application, and, consequently, in movement velocity (Tillaar and Ettema, 2009). Subsequently, the external implement (e.g., a weighted barbell) will decelerate to stop the movement at the end of the concentric phase. Therefore, from a practical standpoint, the continued acceleration given as the “rationale” for the use of elastic bands (or alternative forms of variable resistances, such as adding light or heavy chains) in conjunction with free weights may better reflect the continued acceleration required in the majority of sport tasks (Anderson et al., 2008; Joy et al., 2016; Loturco et al., 2022d; Soria-Gila et al., 2015), thus allowing for greater transference to sport-specific performance (Cormie et al., 2011; Loturco et al., 2022d). Coaches should take into account the specific, and perhaps unique, mechanical aspects of VRT when designing resistance training sessions for their athletes in many sports. As mentioned earlier, the potential benefits of optimizing strength adaptations over short periods may be a crucial factor when selecting training methods in modern sports, where time constraints are always an issue. This could be an obvious and interesting advantage of incorporating VRT into their regular training routines.

## Eccentric and Concentric Training (and Machine-Based Training)

Among the different resistance training modalities, 63%, 58%, and 53% of Brazilian Olympic sprint and jump coaches reported using eccentric, concentric, and machine-based training, respectively (Loturco et al., 2023b). Regarding eccentric training, its prescription mainly relies on the premise that this training method has been shown to: (1) improve stretch-shortening cycle function, and strength, power, and speed performance in different athletic populations (Douglas et al., 2017b; Mcneill et al., 2019); (2) reduce injury risk and its incidence in sprint-based sports (Križaj et al., 2022) where the hip extensor muscles are heavily (and eccentrically) taxed (Kenneally-Dabrowski et al., 2019); and (3) elicit important specific and positive morphological along with structural adaptations (e.g., changes in muscle hypertrophy and architecture and tendon properties) (Križaj et al., 2022; Timmins et al., 2021).

For example, in a laboratory-based study, a 6-week eccentric training protocol (i.e., eccentric-only knee flexor training executed on an isokinetic dynamometer) resulted in important architectural alterations of the biceps femoris long head (i.e., increased fascicle length) compared to a concentric-only program (Timmins et al., 2016). In a more applied research setting, a sample of professional Australian football players completed either a flywheel or a Nordic hamstring exercise program during the pre- and in-season periods (Timmins et al., 2021). Those authors observed greater biceps femoris long head fascicle lengths post-intervention in both groups, confirming the effectiveness of this training method in inducing morphological alterations at the muscular level. These changes are noteworthy (and desired) since increased fascicle lengths may “protect” the hamstrings by enhancing their capacity to withstand stretching and decreasing sarcomere strain during high-speed running activities in which this muscle group is actively lengthened (Gérard et al., 2020). Nevertheless, caution is necessary when implementing eccentric exercises (e.g., Nordics) due to the muscle damaging nature of this type of muscle contraction (i.e., a phenomenon described as “exercise-induced muscle damage”; [EIMD]) if training is not properly prescribed, which may result, for example, in elevated levels of delayed onset muscle soreness (DOMS) (Proske and Morgan, 2001).

Another important consideration concerning eccentric training relates to its influence on strength-power-speed performance (Douglas et al., 2017b; Križaj et al., 2022; Mcneill et al., 2019). For instance, De Hoyo et al. (2014) concluded that a 10-week eccentric-overload training protocol (i.e., squat and leg-curl exercises using flywheel ergometers) applied to a sample of young soccer players resulted in meaningful improvements in vertical jumping and sprinting abilities when compared to a control group that only performed their regular soccer technical-tactical training. Moreover, greater eccentric strength in knee flexors and knee extensors has been associated with faster completion times and superior deceleration abilities during aggressive directional changes (i.e., 180º COD task) (Jones et al., 2016). This may be explained, at least in part, by the fact that considerable braking forces and pronounced eccentric muscle actions are required to reduce the center of mass velocity during sharp COD maneuvers (Dos’ Santos et al., 2018). Thus, aligned with this idea, a compelling body of evidence supports the effectiveness of eccentric training strategies in improving COD ability (Chaabene et al., 2018).

With respect to concentric training, a comprehensive study that assessed 303 elite male and female athletes from various sports (i.e., soccer, futsal, rugby, and handball) observed that those capable of producing greater mechanical power in the concentric phase of the half squat and jump squat exercises displayed, overall, superior sprint, jump, and COD abilities (Loturco et al., 2018b). It is important to note that the half squat and the jump squat were “pure” concentric actions, as players were instructed to pause at the bottom of the squat (i.e., thighs at ~90°) before executing the upward phase of the lift as fast as possible, thus eliminating the contribution of the eccentric portion of the movement. Hence, it appears that “pure” concentric force and power generating capabilities are important determinants of linear and multidirectional sprint and vertical jump performance (Loturco et al., 2018b). In addition, several studies (De La Cruz et al., 2023; Pareja-Blanco et al., 2016; Randell et al., 2011; Zhang et al., 2022) have demonstrated the effectiveness of “concentric-oriented” resistance training programs (e.g., velocity-based training programs) in improving physical performance measures (e.g., sprint speed or vertical jump measures) in trained individuals. It is not surprising then that elite sprint and jump coaches utilize this training method within their programming.

It is also worth noting that half of the Brazilian coaches (53%) indicated using machine-based training as part of their resistance training routines. This choice may be related to the fact that, as reported by Healy et al. (2021), sprint coaches usually select exercises based on two main aspects: (1) the muscle groups involved (i.e., whether or not they are important for sprinting); and (2) practicality (i.e., whether they are easy to implement and execute). Therefore, machine-based exercises may be a reasonable option given that they “check both boxes”. Moreover, recent research found no differences in dynamic and isometric strength, hypertrophy, vertical jump adaptations (Haugen et al., 2023), and muscle architecture changes (Hernández-Belmonte et al., 2023) when comparing the effects of free-weight vs. machine-based strength training, thus indicating that both training modalities may be effective for improving performance. In essence, it appears that choosing one over the other is down to individual preference, but authors also mention that it is likely that a combination of both strategies would be ideal (Haugen et al., 2023). Based on the previous concepts and considering the most determinant muscle groups in sprinting (i.e., hip and knee flexors and extensors, and ankle plantar flexors) (Novacheck, 1998), equipment like the leg-extension, leg-curl, multi-hip, hip-thruster, or standing calf-raiser machines can be incorporated into the resistance training programs of elite and young athletes engaged in high-intensity running-based sports, particularly when combined with traditional and ballistic free-weight exercises (e.g., squats, jump squats, lunges or deadlifts).

In summary, from a practical perspective, coaches are recommended to utilize and combine a variety of resistance training methods in their programming. Eccentric training may be preferred for injury prevention, to promote desired morphological alterations (i.e., positive adaptations in muscle architecture or hypertrophy), or to enhance an athlete’s deceleration and COD capabilities. However, its application close to important training sessions or competitions should be carefully considered due to the potential damaging nature of eccentric muscle actions. Finally, concentric and machine-based training may be complementary, according to individual preferences and practicality, aiming to improve strength, power, and speed performance throughout the competitive season.

## Isometric Training

Although isometric training is becoming increasingly popular within the scientific and coaching communities, only 37% of Brazilian Olympic sprint and jump coaches reported using this method on a regular basis with their athletes (Loturco et al., 2023b). This might be explained by the fact that sprinting and jumping are dynamic activities that mainly involve the use of the stretch-shortening cycle (Novacheck, 1998), which means that isometric contractions are not as determinant for these specific skills (Loturco et al., 2023b). Nevertheless, practitioners should not overlook the potential benefits of adding isometric strength training to their regular programming routines.

Bimson et al. (2017) investigated the effects of a 6-week maximal isometric knee extension training intervention (i.e., 3-s contractions at 7 joint angles: 108º, 96º, 78º, 60º, 42º, 24º, and 12º) performed once a week alongside soccer-specific training and observed significant improvements in vertical jump height when compared to a control group. Similarly, Lum et al. (2023) concluded that the inclusion of long-term (i.e., 24 consecutive weeks) or periodic (i.e., two 6-week periods over 24 weeks) isometric training resulted in significantly greater gains in linear sprint performance compared to a control group in a sample of floorball athletes. Furthermore, when performed as fast as possible, isometric contractions have also been reported to result in meaningful improvements in explosive strength, as assessed by the means of the rate of force development (RFD) (Lum and Barbosa, 2019). Therefore, recent research seems to support the use of isometrics to improve athletic performance measures, mainly when prescribed as a complement to sport-specific training or combined with dynamic strength-power exercises (Lum and Barbosa, 2019).

Notably, isometric contractions have been shown to have a lower metabolic cost than dynamic contractions (Newham et al., 1995). The reduced energy demand of isometric training suggests that lower levels of post-exercise fatigue may be expected (Lum and Barbosa, 2019). From an applied standpoint, this training method could be considered an alternative to dynamic strength training that coaches can utilize during congested periods of the season when recovery is paramount. A suitable strategy could be to replace some dynamic exercises (e.g., half squat) with an isometric variation of the same exercise, or to reduce the total number of dynamic contractions during the workout (i.e., prescribe less sets and repetitions), replacing them with short, high-intensity isometric contractions. A crucial aspect to consider, nevertheless, is the concept of “joint-angle specificity” as it pertains to isometric training. This concept arises from the multiple observations that post-isometric training adaptations seem to be maximized around the joint angles used during the training sessions (Kubo et al., 2006; Lanza et al., 2019; Lum and Barbosa, 2019; Noorkõiv et al., 2014). Hence, practitioners are recommended to prescribe isometrics at different joint angles, especially when the main objective is to improve athletic (dynamic) performance (Lum and Barbosa, 2019).

A recurrent theme highlighted in the scientific literature is the possible existence of two different types of isometric actions: (1) “holding isometric muscle actions” (HIMAs), also termed “yielding isometrics”, which consist of an isometric action employed to resist an eccentric (lengthening) contraction (i.e., isometrically maintaining/holding a position with or without external resistance, as in a wall-squat); and (2) “pushing isometric muscle actions” (PIMAs), also called “overcoming isometrics”, which essentially correspond to an isometric action in which an individual pulls/pushes against an immovable object, attempting to generate movement (e.g., an isometric mid-thigh pull) (Garner et al., 2008; Schaefer and Bittmann, 2017). Theoretically, HIMA is an eccentrically-biased exercise since the aim is to prevent muscle lengthening. In contrast, PIMA is more closely related to a concentric contraction (i.e., the intent is to move a fixed load through a muscle shortening action) (Garner et al., 2008). Based on the above-mentioned, the former may be recommended to enhance eccentric strength and deceleration capabilities, while the latter would be more suitable for developing concentric-based strength and acceleration abilities. However, this hypothesis requires further exploration, and future studies should examine the relationships between these two different isometric actions (i.e., HIMA and PIMA) and athletic performance measures, as well as motor tasks. This investigation should focus specifically on activities involving eccentric and concentric contractions, such as horizontal decelerations and accelerations.

Isometric training has also been considered a promising resistance training method from an injury prevention and rehabilitation perspective. As demonstrated by Rio et al. (2015), isometric contractions can have an acute analgesic effect in team-sport athletes (i.e., volleyball players) with patellar tendinopathy, commonly referred to as “jumper’s knee”, as players reported lower pain scores for at least 45 min post-exercise. Furthermore, in another investigation led by the same authors (Rio et al., 2017), the effects of completing a 4-week isotonic muscle contraction protocol (i.e., 4 x 8 repetitions at 80% of 1RM of the leg extension exercise) versus an isometric muscle contraction intervention (i.e., 5 x 45-s holds at 80% of the maximal voluntary contraction [MVC]) were analyzed and compared. The results showed that the latter resulted in a significantly greater decrease in tendon pain during the in-season period in which players continued their regular training and competition schedule. As a consequence, including isometrics before a workout or a competition may enhance performance in athletes with tendinopathy due to the immediate and long-lasting pain relief (Rio et al., 2015, 2017). Finally, another noteworthy aspect is the “protective effect” that isometric muscle actions may have against exercise-induced muscle damage, when performed 2–4 days before intense exercise, as a pre-conditioning activity (Boyd et al., 2023), which reinforces the usefulness of this type of resistance training method during the competitive season.

In conclusion, although not widely prescribed by Brazilian Olympic sprint and jump coaches, isometric training may contribute to improved maximum and explosive strength (i.e., RFD), and jump and sprint performance. In this regard, coaches should primarily consider prescribing PIMA across a range of joint angles, with short duration (i.e., 3–5 s), using maximal or near-maximal contractions (i.e., 80–100% MVC), executed as fast as possible, with a total volume of 30–90 s per session (Lum and Barbosa, 2019). Conversely, if the goal is to take advantage of the isometric exercise-induced analgesia, HIMA should be preferred, incorporating lower contraction intensities (e.g., 70–80% MVC) and longer duration (i.e., ~45 s).

## Complex Training

When it comes to resistance training methods utilized for speed development, complex training (considered a “tertiary method”: methodologies that do not mimic or reflect sprint movements, but provide important stimuli that may lead to positive adaptations in sprint performance) (Zabaloy et al., 2023), is one of the most commonly employed by coaches working at the elite level in different sports (Loturco et al., 2023b; Weldon et al., 2021b). The term “complex training” is used to describe training strategies that alternate movement velocity or loads between sets and/or exercises in the same strength-power training session, with the aim of optimizing slow and fast force production (Cormier et al., 2022). A recent literature review (Cormier et al., 2022) identified different complex training strategies, with complex contrast training (CCT) being the most typically prescribed. In brief, CCT consists of alternating high-load/low-velocity and low-load/high-velocity exercises in a set-by-set fashion in the same session (Cormier et al., 2022) and has been reported to lead to positive adaptations in speed-power capabilities, specifically in linear sprinting and COD speed (Cormier et al., 2020; Freitas et al., 2017; Marshall et al., 2021; Thapa et al., 2021).

To date, the mechanisms underlying improvements in athletic performance following CCT are yet to be fully elucidated. Some authors suggest that including a high-intensity conditioning activity (CA) (e.g., half-squat) before an explosive low-load/high-velocity exercise (e.g., a vertical jump), may increase motor neuron excitability and reflex potentiation (Ebben et al., 2004). Other authors propose that completing a CA may elicit a “post-activation performance enhancement” response, believed to occur due to acute variations in muscle temperature, muscle/cellular fluid content, or even to motivational aspects (Blazevich and Babault, 2019; Cormier et al., 2022; Cuenca-Fernández et al., 2017). These responses essentially prime the central nervous system, facilitate muscle activation, and result in a (transient) increase in maximal strength-speed-power performance (Blazevich and Babault, 2019; Cormier et al., 2022; Cuenca-Fernández et al., 2017). Finally, another hypothesis is that the effectiveness of CCT can be explained by its mixed-training approach, where exercises stimulating different areas of the force-velocity spectrum (i.e., high-force and high-velocity portions of the force-velocity spectrum) are performed within the same session (Cormier et al., 2020, 2022; Haff and Nimphius, 2012), potentially improving both slow and fast force expression. Nonetheless, despite ongoing debates about its underlying mechanisms, CCT remains extensively used in practical settings (Ebben et al., 2004; Simenz et al., 2005).

In fact, CCT is the primary strategy used to integrate plyometric exercises within resistance training programs across a variety of sports, including ice hockey (Ebben et al., 2004), basketball (Simenz et al., 2005), rugby (Jones et al., 2016), and soccer (Weldon et al., 2021c), as reported by professional strength and conditioning coaches. Interestingly, the percentage of practitioners in individual sports (i.e., Olympic sprint and jump coaches) that declared prescribing plyometrics as part of CCT was slightly lower (42%) than in team-sports (57%, 60%, 58%, and 52% in ice-hockey, basketball, rugby, and soccer, respectively). This difference is likely attributed to the match-congested schedules and the typically shorter strength and conditioning sessions in sports like soccer (i.e., a typical session lasts around 30–45 min) (Loturco et al., 2022a; Weldon et al., 2021c) compared to track and field events, where the most frequent session duration is between 61 and 75 min in the preparatory period and 46–60 min during the competitive phase of the season (Loturco et al., 2023b). Therefore, it appears that CCT is a suitable and recurrent option in time-constrained contexts, which occur much more frequently in team-sport disciplines.

In terms of programming, responses to CCT interventions are highly individualized, as athletes’ characteristics (e.g., relative strength or the performance level) may affect training outcomes (Cormier et al., 2022). Specifically, research indicates that low training frequencies (i.e., 2 sessions per week) may be sufficient to induce meaningful performance improvements (Freitas et al., 2017). Moreover, in team-sports, greater speed-related adaptations have been shown to occur when submaximal CAs (<85% 1RM) are used (Cormier et al., 2020; Freitas et al., 2017), conceivably due to this loading scheme resulting in: (1) a positive acute potentiation-fatigue relationship that facilitates the execution of the explosive exercise of the contrast pair (Cormier et al., 2022); and (2) greater overall movement velocities during the workout, which can be a potent stimulus to elicit speed-related adaptations (Behm and Sale, 1993; Mcbride et al., 2002). Lastly, when prescribing CCT programs, it is worth noting that although the long-term adaptations to different intra-contrast rest intervals remain largely unknown, different meta-analyses have revealed that rest periods longer than 2 min between the CA and the subsequent exercise seem to lead to superior performance outcomes (Cormier et al., 2020; Freitas et al., 2017). Nonetheless, it should be mentioned that weaker individuals may benefit from longer rest intervals (i.e., ≥ 7–8 min), even though this may not always be “practical” in real training settings. Conversely, shorter rest intervals (i.e., from 2 to 5 min) may be adequate to induce relevant long-term adaptations in stronger individuals. This also highlights the importance of practitioners adjusting these intervals and determining the optimal “intra-contrast rest intervals” tailored to their athletes before implementing a CCT intervention (Cormier et al., 2020; Freitas et al., 2017).

In summary, CCT is a time-efficient method that may be beneficial for speed development, particularly in contexts where the time available for strength-power training sessions is limited (e.g., team-sports or during the competitive period in track and field). The mechanisms underlying the adaptations following CCT are not fully understood, but current evidence indicates that moderate loads (<85% 1RM) and low training frequencies may result in meaningful improvements in sprint performance (Cormier et al., 2020, 2022). This emphasizes the practical and advantageous applicability of this training strategy year-round in both individual and team-sports, encompassing athletes with diverse performance levels and backgrounds, including youth and adult populations.

## Isoinertial Training

During the use of traditional resistance exercises (e.g., squats, deadlifts) equalized loads are employed for both the concentric and eccentric phases of the lift (Murton et al., 2023). Furthermore, during eccentric contractions, muscles act while lengthening due to the applied external resistance that exceeds the momentary force produced by the muscle (Beato, 2020; Douglas et al., 2017a). This mechanical advantage also serves as the basis for incorporating flywheel exercises, in which the eccentric phase experiences an overload due to the inertia accumulated during the concentric phase, provided the latter is executed with maximal intent and effort (Maroto-Izquierdo et al., 2017). Under these circumstances, it seems reasonable for strength and conditioning coaches in numerous sports to incorporate isoinertial exercises into their training programs. Despite these potential advantages, specifically, only 16% of Brazilian Olympic sprint and jump coaches declared using inertial exercises with their athletes (Loturco et al., 2023b). The low percentage of isoinertial training usage among Brazilian coaches is likely related to the absence of isoinertial devices in Brazilian training facilities, owing to the expensive cost and unavailability of this type of equipment (Loturco et al., 2022a, 2023b).

Indeed, track and field athletes, such as sprinters and jumpers, may benefit from the utilization of eccentric overloading, particularly for the lower limbs. In this context, there is a well-established mechanistic link between running mechanics and hamstring strain (Bramah et al., 2023), which typically occurs during the late-swing phase just before the touch-down, with the biceps femoris long head being the most commonly affected muscle (Zabaloy et al., 2023). Therefore, achieving long-term increases in capacities such as power, sprint-specific endurance, and technique, along with simultaneous enhancements in sprinting performance, requires maximizing adaptations in muscle contractile machinery while minimizing the risk of overreaching, excessive fatigue, and injuries in highly-specialized athletes (Agudo-Ortega et al., 2023; Haugen et al., 2019). Isoinertial exercises (e.g., flywheel exercises), with their recognized effectiveness in generating a more demanding eccentric phase due to the increased mechanical load required to absorb the kinetic energy stored in the flywheel and gradually decelerate this load, may be an important resource in this regard (Beato, 2020; Beato et al., 2019; Douglas et al., 2017a).

Isoinertial training using flywheel devices has been shown to be effective in improving jumping, linear sprinting, and COD abilities in soccer players (De Hoyo et al., 2014; Tous-Fajardo et al., 2015). Likewise, Murton et al. (2023) reported that flywheel and traditional resistance training are similarly effective in improving lower-body strength and power qualities (i.e., jump tasks) in male academy rugby union plyers. In recent survey-based studies (Loturco et al., 2022a; Weldon et al., 2021a; Zabaloy et al., 2022b), authors reported that isoinertial devices were regularly used by 25%, 15%, and 6% of coaches and practitioners in soccer, cricket, and rugby union, respectively. It is important to note that despite the advantages and positive points discussed earlier, there are some disadvantages associated with the use of these devices (Loturco et al., 2022a, 2023b) including the expensive costs and difficulties observed in precisely controlling and prescribing the adequate training load (Beato, 2020). The mentioned difficulties may hinder or, at least, reduce the possibility for coaches to incorporate isoinertial resistance training into their daily practices. Furthermore, considering that isoinertial devices can be used to mimic traditional strength-power (e.g., squats, deadlifts) or even machine-based exercises (e.g., lying leg curls), it could be argued that the reported positive effects are also dependent on the type of exercise and not solely on the technology being applied (De Hoyo et al., 2014; Tous-Fajardo et al., 2015).

Overall, as indicated by Beato (2020), practitioners should consider two important factors when implementing isoinertial training with their athletes: (1) current literature suggests that flywheel training is a viable alternative to traditional weight training methods, although there is not enough evidence to suggest that it is superior among elite athletes; (2) isoinertial training produces different physiological responses to other types of resistance exercise or resistance training methods. Hence, it seems reasonable to incorporate both flywheel and traditional resistance training into typical training programs to optimize the benefits of these types of training. Finally, and equally importantly: if coaches lack the resources to acquire or use appropriate isoinertial devices, it may be feasible and reasonable to apply eccentric training overloads through simple yet similarly effective exercises and drills, with or without the use of low-cost equipment (e.g., Nordic hamstring, Swiss ball hamstring curls, etc.) (Alonso-Fernandez et al., 2017; Guruhan et al., 2021).

## Resistance Training Exercises

The resistance training exercises most frequently used by Brazilian Olympic sprint and jump coaches in their daily practices are listed in Table 2(Loturco et al., 2023b). This table serves as a brief introduction to the comprehensive description and analysis of each exercise, including their variations and potential utilization, as presented in this manuscript.

## Squat and Its Variations

Alongside Olympic weightlifting and its derivatives, squats and their variations were the only exercises present in the five different lists prepared by the Brazilian Olympic sprint and jump coaches. Possible explanations for this preference and importance are linked to the confirmed effectiveness of this exercise type and its close relationship with acceleration and top-speed performance (Loturco et al., 2018a; Wisløff et al., 2004). Additionally, the applicability and ease of executing squats, and lastly, the numerous variations that can arise from this traditional resistance training exercise certainly contribute to its great popularity among practitioners from many sports (Loturco et al., 2015b; Weldon et al., 2021b). Several studies have compared the effects and biomechanical characteristics of squats performed at various depths (e.g., shallow versus full squats), loading ranges (e.g., heavy versus light loads) and positions (e.g., back versus front squats; high versus low barbell squats), revealing valuable insights into these aspects (Contreras et al., 2015; Goodin, 2015; Gullett et al., 2009; Martínez-Cava et al., 2019; Yavuz et al., 2015). Concerning kinetics and kinematics, Goodin (2015) demonstrated that high barbell back squats tended to produce greater peak force, power, and velocity across a comprehensive range of loads (i.e., 20–90% 1RM), whereas low barbell back squats tended to generate a larger impulse, especially above 80% 1RM. According to that author, a potential reason for the superior impulse in the lower barbell back squat is derived from the slower (relative) barbell velocity. In other words, subjects take a longer time to complete this movement, providing them with more time to apply force compared to the high barbell back squat. Muscle activity and kinematics between front and back squats have also been another important point of discussion in sport science (Contreras et al., 2015; Gullett et al., 2009; Yavuz et al., 2015). Generally speaking, in comparison with the front squat under maximum loading conditions, the back squat exhibits a greater trunk lean, without presenting meaningful differences in the knee joint kinematics across the entire range of motion, which may be interesting for preventing and reducing possible lumbar injuries and pain (Yavuz et al., 2015). In addition, despite requiring lighter loads, the front squat can result in higher electromyography activity in the vastus medialis and lead to less compressive forces and lower knee extensor moments on the knee joint (Gullett et al., 2009; Kaardal, 2019). Therefore, this squat variation becomes a viable alternative for focusing on the improvement of knee extensors or for reducing the rate of injury and the incidence of pain in the lumbar region, particularly during heavy loading training programs (Gullett et al., 2009; Kaardal, 2019; Yavuz et al., 2015).

Other mechanical relationships are also affected by the extent of the range of motion across different squat modes. Accordingly, increases in squat depth considerably alter the load-velocity and the load-power relationships among different depths of squat (i.e., half, parallel, and full back squat) (Martínez-Cava et al., 2019). Martínez-Cava et al. (2019) showed that for shorter ranges of motion (e.g., half squat versus parallel squat), athletes were required to lift heavier loads to achieve the same loading magnitude (% 1RM); thus, the power output produced during half squats was substantially higher than that produced in parallel squats. An interesting finding of that study is that there was a proportional reduction between the mean concentric displacement among the three squat variations (i.e., full versus parallel squat: −9.9 cm or 15.5%; full versus half squat: −7.6 cm or 32.6%), whereas there was a disproportional increase in the 1RM strength (i.e., full versus parallel squat: −7 kg or 8%; parallel versus half squat: −37.1 kg or 39.3%). For those authors, this significant increase in the 1RM value for the half squat was related to the muscle moment arms and specific joint angles achieved during the starting position of this squat variation. Indeed, the highest position adopted during the initial portion of the half squat exercise (i.e., smaller ranges of motion compared to parallel and full squats) prevents athletes from reaching a sticking region (described as a “poor biomechanical region”, where the capacity to apply force is compromised) (Larsen et al., 2021). Of course, these differences should influence the choice of squat variations, which should be based not only on sport specificity (i.e., movements and tasks that athletes regularly perform in their sports), but also on their ability to properly execute the three squat exercises. It is essential to bear in mind that, although deeper squats are generally safe, they also demand better execution techniques.

**Figure 2:**
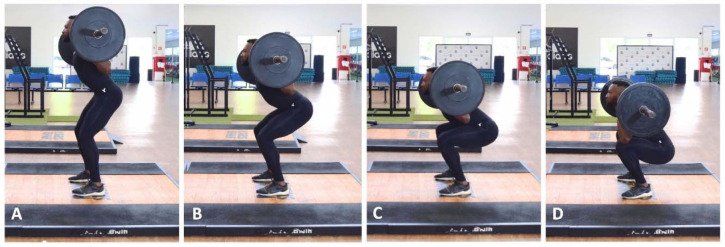
Squat exercise with its variations: (A) shallow squat; (B) half squat; (C) parallel squat; (D) full squat.

Finally, and especially for sprinters and jumpers, shallow squats (i.e., ~0–60° of knee flexion) (Figure 2) appear as one of the most commonly used and useful exercises by track and field coaches (responses collected from open-ended questions; Loturco et al., 2023b). This is because, as well as other types of shallower squats (i.e., squats with smaller ranges of motion such as the half squat), shallow squats allow athletes to utilize heavier loads and move these loads faster (compared to deeper squats) (Larsen et al., 2021; Martínez-Cava et al., 2019). This substantially increases the velocity and the amount of vertical force application in a crucial sprinting position for sprinters and jumpers during the transition from the drive to the top-speed phase (i.e., during this phase, athletes should maintain a more upright position, and it is essential that the foot strike occurs just ahead of the centerline of the body) (Jeffreys, 2013). In fact, elite sprinters and jumpers, due to their high levels of strength, power, and leg stiffness, typically require minimal contact times (and minimal flexion in the angles of the ankle, the knee, and the hip) when sprinting (Jeffreys, 2013). In contrast, “poorer sprinters” (i.e., less specialized sprinters) tend to exhibit excessive flexion in the ankle, the knee, and the hip when touching the ground at higher speeds. This, in turn, concomitantly increases braking forces and contact time, thereby reducing running speed (Jeffreys, 2013). In summary, front, back, shallow, half, parallel, and full squats are widely used forms of squat exercise, that are not limited to track and field disciplines, but extend to a wide variety of sports.

As general and less detailed rules, it is important to emphasize that: (1) deeper squats elicit superior adaptations in knee extensor muscles and leg lean body mass compared to shallower squats (Bloomquist et al., 2013); (2) when analyzing EMG activity across distinct squat depths (e.g., half, parallel, and full squats), it becomes evident that the contribution of the gluteus maximus during the concentric phase of the lift is considerably higher in full and parallel squats compared to shallower movements, such as half and shallow squats (Caterisano et al., 2002); (3) despite requiring lighter loads to achieve the same relative loading range (i.e., % 1RM), front squats may be utilized to prevent lumbar injuries and pain, while exerting less compressive forces on the knees (Gullett et al., 2009; Kaardal, 2019). Three final and key points to consider are that: (1) during the concentric portion of the lift, athletes will achieve the peak velocity progressively earlier in shallower squats—a mechanical behaviour that can be observed, for example, when comparing half, parallel, and full squats under maximum loading conditions (Martínez-Cava et al., 2019); (2) increases in the range of motion (i.e., deeper squats) result in longer moment arms at the knee and hip joints, thus activating larger portions of the front thighs and gluteus muscles (Bryanton et al., 2012; Kaardal, 2019) (this can be particularly important, for example, for coaches interested in developing acceleration- or jumping-related capabilities); (3) shallow squats allow athletes to lift heavier loads at higher velocities, consequently producing greater levels of vertical force in a more upright position. This approach is more similar to the body position adopted during top-speed phases and may be highly recommended for coaches aiming to enhance the top-speed qualities of their athletes. Practitioners from various sports can use this information to choose the most suitable squat variations for their athletes.

## Olympic Weightlifting and Derivatives

In a broader perspective, weightlifting can be defined as a competitive sport that requires athletes to lift a maximal amount of weight in the snatch and the clean and jerk (Morris et al., 2022). The latter two major exercises are further divided into more simplistic exercise regressions: “pulling”, “catching”, and “overhead” derivatives. In the pulling versions, the catch phase of the lift is removed, whereas for the catch derivatives, the depth at which the barbell is caught is altered so that the top of the thigh is above parallel (i.e., “power versions”). These catch derivatives can also be initiated from a variety of positions, such as the floor, knee, hang or mid-thigh positions (Comfort et al., 2023). Importantly, athletes from different sports may benefit from weightlifting pulling derivatives due to the reduced technical complexity compared with catching derivatives. The exclusion of the catch phase reduces the relative complexity of pulling derivatives, making these derivatives naturally easier to teach and learn (Comfort et al., 2023). Another factor worth considering is related to the possibility of applying a wide range of loads (e.g., from 30% to 140% 1RM) in the “pull” compared to “catch” variations (Suchomel et al., 2020). Catching derivatives, on the other hand, are more complex to execute and do not allow for the use of supramaximal loads (Comfort et al., 2023). Nevertheless, authors have already reported some important benefits, such as postural strength, coordinated loaded triple extension and flexion of the knee, hip, and ankle joints, providing a “load acceptance” stimulus comparable to that found during a jump landing, along with the co-contraction of the spinal stabilizing muscles (Comfort et al., 2023).

When Brazilian Olympic sprint and jump coaches were asked whether they used Olympic weightlifting and associated derivatives in their resistance training programs, 79%, 58%, and 47% of coaches reported using the snatch, the clean, and the clean and jerk, respectively (Loturco et al., 2023b). Only a minority of these coaches (< 16%) declared using the power clean or the power snatch or responded that they did not adopt these techniques (5%) (Loturco et al., 2023b). These outcomes reinforce the notion that this training strategy has a very important place in terms of the program design and programming during the training process of elite track and field athletes. Notably, the low number of coaches that declared using weightlifting derivatives is somewhat surprising due to the fact that these variations may allow coaches to use simpler and easy-to-coach movements. In fact, practitioners should integrate Olympic weightlifting, traditional strength-power training, and plyometrics into a more holistic and comprehensive training routine when attempting to enhance athletic performance in other sports (Comfort et al., 2023). Specifically, Olympic sprinters and jumpers may benefit greatly from developing rapid force production, given the maximal or near maximal-force production and velocity observed throughout the entire range of motion of these movements compared to traditional resistance training (e.g., deadlift, back squat) (Crenshaw et al., 2023). Similarly, Olympic weightlifting and its derivatives, along with the squat and its variations, were the two most popular exercises in other field-based team-sports, such as (international) soccer, rugby, cricket, and basketball (Simenz et al., 2005; Weldon et al., 2021a, 2021c; Zabaloy et al., 2022b). In this regard, the latter clearly emphasizes the importance that coaches give and devote to weightlifting and derivatives during the resistance training prescription. Nonetheless, there are several aspects that need to be analyzed when selecting and prescribing these exercises: (1) the technical competency of athletes to execute the lifts, individually and as a group; (2) the previous experience of athletes with these techniques; (3) the time needed to properly teach these movements to athletes, ensuring they go through a progressive educational process from early childhood; and (4) the fact that, up to the present day, there is no clear evidence of the superiority of Olympic weightlifting over traditional resistance training involving different types of strength-power exercises (Berton et al., 2022; Crenshaw et al., 2023; Morris et al., 2022).

Given the time-consuming aspects inherent in the teaching process of weightlifting, coaches working with large groups of athletes on a daily basis (i.e., team-sports) may find it challenging, for example, to properly coach and teach the snatch and the clean and jerk during the annual season. On the other hand, pulling variations may be better suited to address the challenges faced by practitioners. This is not only due to the aforementioned reasons, but also because it has been reported that implementing a wide range of loads (i.e., from 30% to 140% 1RM) with pulling derivatives results in superior adaptations in relative strength, sprint speed, and COD performance compared to sub-maximally loaded weightlifting catching and pulling derivatives (Suchomel et al., 2020). Likewise, Morris et al. (2022) reported that weightlifting was most effective for improving weightlifting performance, while limited differences existed between this training method, traditional resistance training, and plyometrics in terms of increasing strength and linear and multidirectional sprint speed. As a result, coaches and practitioners in individual and team-sports must carefully consider and select the most appropriate exercises to better prepare their athletes based on the adaptations that will be induced (including acute and long-term effects) and how these choices may affect athletic performance. To date, no clear evidence exists regarding the superiority of weightlifting (i.e., clean and jerk, and snatch) and derivatives like the power versions over traditional strength-power exercises in relation to sprint performance (Crenshaw et al., 2023; Morris et al., 2022). In summary, while authors recommend integrating weightlifting techniques into a traditional strength and conditioning program (Suchomel et al., 2020), practitioners should also consider the challenges and difficulties of prescribing and utilizing Olympic weightlifting and its derivatives (e.g., complexity, technical competency, proper coordination). Therefore, depending on the athletes’ training background, physical and technical demands, and objectives of the sport they are involved in, coaches may decide how, when, and why they should (or should not) employ weightlifting techniques with their athletes. Additionally, they need to determine which derivatives are more appropriate and specific to meet the needs of their athletes at a particular phase of the season (Comfort et al., 2023; Suchomel et al., 2015).

## Ballistic Exercises

The term ballistics derives from the Greek “ballein” (Vesilind, 2006), which means to throw, and it also refers to projection. Therefore, in practical terms, specifically in resistance training, ballistic exercises include actions like throwing or projecting the body in vertical or horizontal directions (e.g., jump exercises) (Loturco et al., 2023d). According to Cormie et al. (2011) ballistic exercises preclude any deceleration phase during the concentric portion of the lift, requiring athletes to apply force consistently “throughout the entire range of motion”, up to the point of projection (i.e., jumping takeoff). This mechanical characteristic (i.e., 100% accelerative movement) makes ballistics, and especially the jump squat, one of the preferred and most popular exercises for track and field coaches. In fact, Healy et al. (2021) reported that the jump squat was the most commonly prescribed ballistic and the second most regularly used exercise among qualified sprint coaches, even when compared with other traditional, ballistic, plyometric, and unweighted jump-type movements. A similar trend was observed by Bolger et al. (2016) in a study using a survey-style approach, which interviewed seven high-level sprint coaches about their perceptions and views on resistance-based training interventions. In fact, for those sprint coaches, the squat jump was the most frequently used form of loaded barbell squat exercise (Bolger et al., 2016). This high degree of importance was also evident in the original survey conducted with Brazilian Olympic sprint and jump coaches (Loturco et al., 2023b), who included ballistics (especially, loaded jump squats; responses collected from open-ended questions) in four out of the five primary lists of strength-power exercises most commonly prescribed in their programs, and it was reported to be regularly used by ~50% of those coaches. The preference for ballistic movements is not exclusive to track and field coaches; this tendency may also be observed in other sports (Cormie et al., 2011; Cronin et al., 2001; Newton et al., 1999). In general, these exercises can be used in isolation, as part of more comprehensive resistance training sessions or even as movements integrated within complex training routines (e.g., executing a heavy-load dynamic exercise before performing a light-load jump squat) (Weldon et al., 2021b).

The choice of ballistic movements is not only related to their strong relationships and positive effects on a series of performance-related variables, but also to their mechanical characteristics (Cormie et al., 2011; Loturco et al., 2023d). As mentioned earlier, ballistics are entirely accelerative, requiring athletes to accelerate across the complete range of motion, which, in turn, brings some potential advantages to these exercises (Cormie et al., 2011; Cronin et al., 2001; Loturco et al., 2023d; Newton et al., 1996). For Loturco et al. (2023d), these mechanical advantages are clear and easily explained, especially when the main objective of training is to improve physical and technical capabilities that directly depend on qualities such as maximum acceleration and speed (e.g., sprinting- and jumping-related actions). Firstly, it is worth noting that correlations among bar-power output, linear sprint and jumping abilities tend to be stronger for ballistics compared to traditional squats (r = 0.68–0.72 and 0.64–0.67, respectively, for average mean and peak power values), confirming the sport-specific nature of these exercises (Cormie et al., 2011; Loturco et al., 2023d). In addition, previous studies (Loturco et al., 2016, 2021b) have shown that the transfer effect coefficient (TEC; i.e., the process by which improving performance in a given exercise can positively impact performance in sport-specific tasks) (Issurin, 2013) is greater for ballistic movements compared to their non-ballistic counterparts (e.g., loaded jump squats vs. back squats). A special focus should be given to an interesting study on transference, which revealed that the loaded jump squat (executed with the load that maximizes power output) was superior to Olympic push-press in increasing linear and COD speed, as well as vertical jumping ability in elite young soccer players (Loturco et al., 2016). As those authors suggested, the superiority of loaded jump squats might be related to their mechanical characteristics that closely resembled the segmental triple extension (without decelerating) exhibited during the sprint strides, particularly over the top-speed phases (Loturco et al., 2016, 2023d).

**Figure 3:**
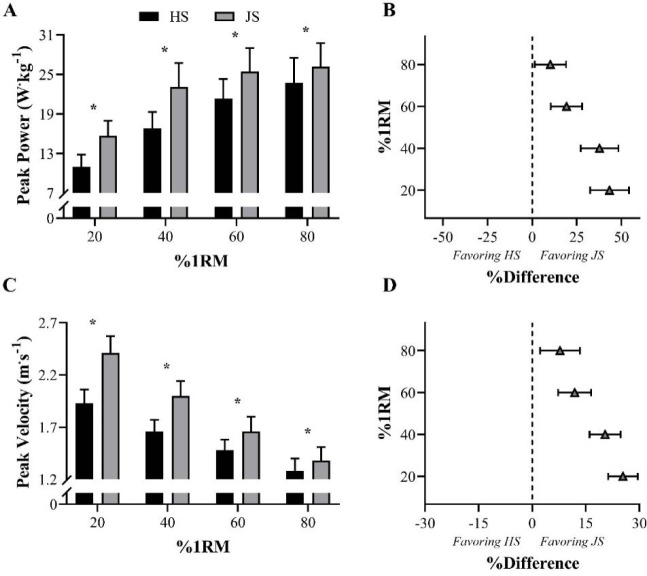
Comparison of peak power and peak velocity between half-squat (HS) and jump squat (JS) exercises across different loads. * Indicates significant differences between the two exercises (*p*< 0.05). Panels A and C display means and standard deviations, while panels B and D show percentage differences and 95% confidence limits. %1RM = percentage of HS 1RM (Loturco et al., 2023d).

**Figure 4:**
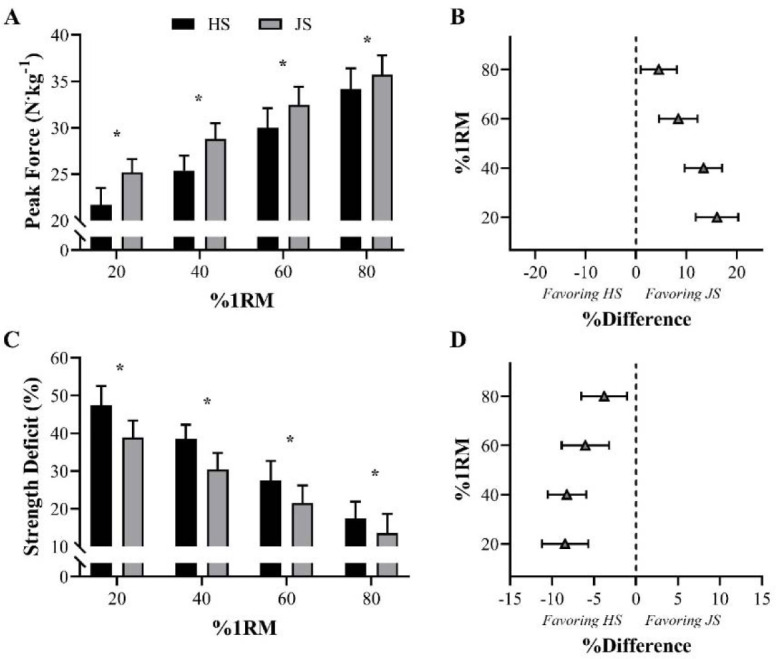
Comparison of peak force and strength deficit between half-squat (HS) and jump squat (JS) exercises across different loads. * Indicates significant differences between the two exercises (*p*< 0.05). Panels A and C display means and standard deviations, while panels B and D show percentage differences and 95% confidence limits. %1RM = percentage of HS 1RM (Loturco et al., 2023d).

Despite the apparent advantage of ballistic movements in improving athletic performance (Cormie et al., 2011; Kraemer and Newton, 2000; Newton et al., 1996, 1999; Wilson et al., 1993), Loturco et al. (2022c, 2023d) indicated that, when performed under heavy loading conditions (i.e., ≥ 80% 1RM), the effectiveness of this type of exercise may be undermined. This is because at intensities ~ 80% 1RM (specifically for the loaded jump squat) the concentric portion of the lift is almost entirely propulsive. As a result, when using these loads, athletes do not have to decelerate at the end of the concentric phase to return to zero velocity, since bar-velocity is too low (Loturco et al., 2022c). This mechanical effect not only does not hamper (or even preclude) the subsequent jump task, but also reduces the differences in kinetic and kinematic output between ballistic and traditional (non-ballistic) exercises (Loturco et al., 2022c, 2023d). From a general perspective, the positive differences (i.e., higher peak power, peak force, and peak velocity) in favor of the ballistic jump squat decrease progressively with increasing loads (i.e., from 20 to 80% 1RM) (Loturco et al., 2023d; Pérez-Castilla et al., 2020) (Figures 3 and 4). In contrast, under light-to-moderate loading conditions (i.e., 20–60% 1RM), force and power production are substantially higher in ballistic exercises. Lighter loads also present another key advantage when comparing ballistic with non-ballistic exercises: under light-loading conditions, ballistics exhibit lower levels of strength deficit (Figure 4C) (i.e., a variable that represents the difference between the force produced at the 1RM and any other submaximal force value) (Gonzalez-Badillo et al., 2017; Loturco et al., 2021c; Zabaloy et al., 2022a). In other words, at these intensities, athletes are able to apply higher levels of force (at higher velocities) against submaximal loads, including “unloaded conditions”. This mechanical aspect, with its potential implications, offers a range of advantages and benefits for athletes of multiple sports who have to apply force, as fast as possible, against their own body weight, in order to jump higher, sprint faster, or execute any sport-specific actions at high-velocities. Therefore, coaches from various sports are highly encouraged to systematically incorporate ballistic exercises into their training routines, bearing in mind that these exercises may preserve their mechanical characteristics and be more effective under light- to-moderate loading conditions (when compared to non-ballistic exercises) (Cormie et al., 2011; Loturco et al., 2022b, 2023d; Mcbride et al., 2002).

## Hip Thrust

The hip thrust is included in four out of the five principal lists of resistance exercises prescribed by Brazilian Olympic sprint and jump coaches (Loturco et al., 2023b). This exercise is regularly utilized by ~30% of Olympic sprint and jump coaches (Loturco et al., 2023b) for a simple reason declared by an expert sprint coach in the Healy et al.’s study (2021): “the hip thrust strengthens the glutes, which are the primary hip extensor”. Indeed, the primary and secondary hip extensors (i.e., gluteus maximus, hamstrings, hamstring part of adductor magnus, adductors and posterior fibers of gluteus medius and minimus, along with some muscles that act as posterior vertebral stabilizers, such as the erector spinae) tend to be highly recruited during the execution of hip thrusts (Contreras et al., 2011). With a focus on antero-posterior force production, this exercise can optimize strength development production at the “end-range of hip extension in the gluteus maximus, thereby improving its relative contribution to the hamstrings during hip extension” (Contreras et al., 2011)–an essential aspect for sprinters (Andersen et al., 2018; Williams et al., 2021). However, the widespread use of the hip thrust among sprint, jump, and track and field coaches from various disciplines (Healy et al., 2021; Loturco et al., 2023b; Mendez-Rebolledo et al., 2021) is not solely associated with its biomechanical characteristics, but also with its perceived, and confirmed, effectiveness in enhancing athletic performance (Contreras et al., 2017; Dello Iacono et al., 2017, 2018; Neto et al., 2019).

Indeed, the close relationship between hip thrust output and sprint performance, especially during the maximum acceleration phase, was previously observed in a study conducted with elite sprinters (Loturco et al., 2018a). In that study, there was a positive correlation of 0.93 between hip thrust power and 10-m sprint speed. Vallance (2017) found similar results in a study involving recreationally active subjects (males only), showing negative correlations of approximately −0.70, −0.60, and −0.65 between hip thrust strength and 40-yard, 10-yard, and 20-yard split times, respectively. Interestingly, in the Vallance’s study (2017), the correlations observed between hip thrust strength and sprint speed were stronger than those detected between back squat strength and sprint speed. The findings from Williams et al. (2021) may, at least partially, explain these outcomes. With a sample of twelve male team-sport athletes, those researchers demonstrated that the ground reaction force, measured via force plates, was lower in the hip thrust in comparison to the back squat. Nevertheless, peak electromyography activity of the gluteus maximus, assessed in both legs and expressed as a percentage of the maximum voluntary isometric contraction, was higher in the hip thrust than in the back and split squat exercises. According to those authors, “the increased activation of the gluteus maximus during the hip thrust and its relationship with maximal running speed suggests that this movement may be optimal for training this muscle group, compared to the back squat and the split squat” (Williams et al., 2021). Importantly, and specifically in this case, this inference is based on an exercise that is capable of inducing greater gluteus maximus activity and, thereby, results in a significant increase in horizontal force production, which may induce positive transfer to sprint performance (Contreras et al., 2017; Loturco et al., 2018a; Williams et al., 2021).

Abade et al. (2021) reinforced these findings by comparing the effects of adding either “vertical or horizontal force-vector exercises” (i.e., a back-half-squat or a barbell hip-thrust, respectively) to a general strength-training program (e.g., upper limb and core exercises) for youth soccer players. Overall, both training approaches improved sprinting and jumping abilities, but the hip-thrust showed greater transference to 10- and 20-m sprint speed (Abade et al., 2021). Despite differences in study designs, Ribeiro et al. (2019) found similar results when comparing the effects of two distinct training regimens: vertical + horizontal plyometrics versus squat + hip thrust exercises executed at the optimum power load in young male soccer players during the competitive season. After 12 training sessions, it was concluded that both training schemes led to increases in a number of jump- and speed-related qualities. However, the combination of squats and hip thrust exercises performed at their respective optimum power zones (i.e., utilizing loads that maximize power output) was superior in enhancing 30-m linear sprint and COD abilities (Ribeiro et al., 2019). Finally, in a recent study, Freitas et al. (2023) demonstrated that a multi-training protocol, incorporating vertical and horizontal strength-power exercises (including the hip thrust), was superior to a traditional vertical training protocol (e.g. squats = drop jumps) in improving zigzag velocity among highly-trained under-20 soccer players during a short soccer pre-season. A counterpoint can be found in the study by Jarvis et al. (2019), who did not observe a positive transfer of hip thrust strength into improvements in 40-m sprint performance in collegiate level athletes. Nonetheless, it is worth noting that in the Jarvis et al.’s study (2019), collegiate athletes were submitted to a very heavy loading condition during the resistance training sessions (heavy barbell hip thrusts at 85% 1RM), which might have affected the potential adaptations in speed-related abilities, especially for collegiate level athletes. However, this does not mean that the actual effects of hip thrusts on athletic performance do not deserve further investigation, including new and more varied training protocols and loading intensities.

In summary, the current state of the literature, as well as the preferences and perceptions of Olympic sprint and jump coaches, allow us to encourage coaches and practitioners from various sports to incorporate hip thrusts into their regular training routines, if possible, using light-to-moderate or moderate loading conditions. As emphasized, this exercise appears to be more effective and yields superior results when combined with different types or forms of exercises (e.g., squats, jump squats, plyometrics, etc.) (Abade et al., 2021; Freitas et al., 2023; Ribeiro et al., 2019). When used within these guidelines, this practical exercise can serve as a suitable and effective stimulus for inducing positive adaptations in sprint performance.

## Lunge

In general, the alternate forward lunge is a unilateral leg exercise involving substantial knee and hip extensor activity (Jakobsen et al., 2012). Nonetheless, there are many variations to this exercise that could fit into any training program and become a bilateral exercise (e.g., a stationary lunge). It can be performed with barbells, Smith machines, dumbbells or even kettlebells. In the ballistic lunge, a previous study (Jönhagen et al., 2009) showed that the high-velocity jumping forward lunge was associated with higher electromyographic activity in the rectus femoris, biceps femoris, and lateral gastrocnemius compared with its walking version. Similarly, side and forward lunges have been shown to activate the gluteus maximus (Neto et al., 2020) and gluteus medius (Stastny et al., 2015), which are important muscles for controlling the frontal plane motion of the pelvis-hip complex. Consequently, different variations in the lunge exercise may provide distinct adaptations that will affect performance differently. For example, ballistic lunges will modify the kinetic and kinematic variables (e.g., peak forces, peak power, peak velocity) while possibly reducing the strength deficit associated with this variation, compared to the stationary lunge (Loturco et al., 2023d, 2023e). The latter differences were also observed when comparing two variations of the same exercise: a half squat and a jump squat. Recently, as mentioned above, Loturco et al. (2023d) reported that the ballistic version of the squat (i.e., a jump squat) exhibited higher peak force, power, and velocity which were associated with a reduced strength deficit compared to the traditional half squat performed across a comprehensive range of loads (i.e., 20–80% 1RM). In another very recent study (Loturco et al., 2023e), elite youth soccer players who performed a ballistic training program (i.e., jump squats + lunge hops) during a 4-week off-season period (between two consecutive seasons) demonstrated, at the individual level, superior gains in lower-body power compared to a traditional resistance training program comprising only non-ballistic squats and hops. Hence, given the importance of reducing the strength deficit for improving athletic performance, especially in terms of sprint speed and muscle power (Loturco et al., 2021c; Zabaloy et al., 2022a), practitioners may consider these practical applications and potential advantages when deciding which lunge variations to include in their training programs.

Previous research involving Brazilian Olympic sprint and jump coaches revealed that only a minority (n = 2; < 11%) included lunges in their lists of the five most important resistance exercises (Loturco et al., 2023b). This suggests that the lunge is not a prioritized exercise; however, this does not necessarily mean it is entirely excluded or disregarded in the training process.Similar findings were observed in soccer (Loturco et al., 2022a), where authors noted that lunges appeared four times among the four most common types of exercise within the five selected coaching ranks. Conversely, strength and conditioning coaches in rugby (none of whom mentioned this exercise) (Zabaloy et al., 2022b) or cricket (~6% of the coaches) (Weldon et al., 2021a) do not seem to prioritize, or even include, lunges in their resistance training programs. Prescribing training loads may be a challenging task for coaches when utilizing lunges and their variations, which could be one of the reasons that explain the low percentage of practitioners prioritizing the inclusion of this exercise. Nonetheless, authors have previously reported that results obtained from squat testing can provide useful data for determining loads for other exercises, such as lunges or step-ups (Ebben et al., 2008a). Another study, conducted with untrained participants, reported that ballistic lunge exercises performed with moderate loads (~65% RM) showed higher levels of leg muscle activity, of similar or greater magnitude when compared to using heavy-load lunges executed at slower (and controlled) velocities (Jakobsen et al., 2012). In contrast, performing lunges under heavier loading conditions (i.e., 80% 1RM) may provide a different stimulus and assist in the process of achieving greater increases in maximum strength, primarily attributed to both muscle and neural adaptations (Duchateau et al., 2021). Thus, despite its relatively low prevalence in both track and field disciplines and other sports, practitioners should always remember that the lunge is a simple and useful exercise, which can be performed using a variety of equipment, such as dumbbells, barbells, the Smith machine, etc., and under different loading conditions (i.e., light, moderate or heavy) and execution modes (i.e., ballistic and non-ballistic versions). Coaches should take into account the beneficial characteristics of this exercise when designing resistance training programs for elite athletes.

## Calf Raises

The calf raise exercise was reported to be used by 15% of Olympic Brazilian sprint and jump coaches (Table 2). Because of the nature of this exercise (i.e., a single-joint movement), coaches usually prescribe calf raises during the preparatory period (i.e., a general training phase) of the season or during the rehabilitation process after injuries or chronic pain, to promote muscle strengthening, hypertrophy (i.e., morphological changes), and reduce bilateral imbalances (responses collected from open-ended questions; Loturco et al., 2023b). Calf raises are mostly used during the preparatory period since, during this training phase, coaches aim to develop a more general strength adaptation, preparing isolated joints and muscle groups for the later (and more complex) stages of the season, when exercises with higher mechanical stress, impact forces, and technical complexity are implemented. Indeed, considering that during plyometrics and form running exercises (e.g., hurdle jumps and skipping) the gastrocnemius and soleus muscles are highly activated (Ebben et al., 2008b; Roelker et al., 2022), sprint and jump coaches opt to stimulate the joint/muscle groups involved in calf raises, using an integrative approach in periods close to competitions by prescribing predominantly technical drills (i.e., form running) and low-intensity plyometrics. This strategy may also be followed by practitioners from other sports, as during various sport-specific activities, the gastrocnemius and soleus muscles are highly engaged in concentric and eccentric actions, such as those involving sprinting and jumping tasks (Brazier et al., 2014; Pandy et al., 2021).

## Core Exercises

Core exercises are not ranked among the most important ones by Brazilian Olympic sprint and jump coaches (Loturco et al., 2023b). Nevertheless, these exercises seem to play a specific role within the programming of these highly specialized practitioners. According to Kibler et al. (2006), “core” is a term typically used to refer to the trunk muscles as a whole, but it specifically includes the abdominal, back, pelvic floor, diaphragm, hip and gluteus muscles. Their main functions are to: (1) establish an effective connection between the upper and the lower body; (2) generate and transfer forces from the proximal areas of the body to the distal parts during daily and sport activities; and (3) stabilize the spine and the pelvis (Kibler et al., 2006). In recent years, different studies have concluded that core and trunk muscle training might result in significant improvements in measures of athletic performance, such as maximum strength, muscular endurance, balance, linear sprint speed, vertical jump height, and COD speed and agility capabilities (Rodríguez-Perea et al., 2023; Saeterbakken et al., 2022) as well as lumbo-pelvic control (Leong, 2024; Mendiguchia et al., 2021; Mills et al., 2005). Additionally, sport-specific performance (e.g., throwing velocity in handball or driving distance in golf) seems to be positively influenced by core training (Rodríguez-Perea et al., 2023; Saeterbakken et al., 2022).

The inclusion of trunk muscle and core training can be advantageous for sprinters and jumpers from a performance perspective (Rodríguez-Perea et al., 2023; Saeterbakken et al., 2022), but also from an injury prevention point of view, given that altered lumbo-pelvic control has been suggested as a potential risk factor for hamstring strains (Mendiguchia et al., 2017) and upper-body injuries (Laudner et al., 2021). However, a common concern among practitioners is whether these specific exercises should be performed in isolation (i.e., in separate workouts) or in combination with other types of exercise (e.g., in sessions with plyometrics or upper- or lower-body lifts) to optimize their effectiveness. In this regard, Rodríguez-Perea et al. (2023) analyzed the pooled effects of interventions consisting of isolated and combined core training routines and concluded that both approaches could be used to improve balance (i.e., isolated: standardized mean differences (SMD) = 0.67; combined: SMD = 1.67), throwing/hitting velocity (i.e., isolated: SMD = 0.52; combined: SMD = 1.67) and jumping ability (isolated: SMD = 1.01; combined: SMD = 0.86). From an applied perspective, this indicates that practitioners may choose to implement their core training routines in separate sessions or combine them with strength-power exercises or sport-specific activities, according to their particular contexts.

An aspect that stands out when critically analyzing the available literature on core training is that session duration has been identified as a potential moderating factor for improvements in strength, linear sprint speed, and COD speed (Saeterbakken et al., 2022). Specifically, shorter session duration (i.e., ≤ 30 min) seems to result in significantly greater gains in the mentioned physical performance variables when compared to sessions longer than 30 min (Saeterbakken et al., 2022). For example, Brull-Muria and Beltran-Garrido (2021) reported that performing a 20-min core training session before soccer practice, twice per week for eight weeks, resulted in significantly faster 10-m sprint and V-Cut (i.e., a specific COD task) completion times in a group of youth players. This finding may have important implications for sports in which the time available for strength and conditioning practices is limited (e.g., team-sports), since it appears that, even with short session duration (e.g., 15–20 min), significant benefits can be obtained from core training.

To summarize, the literature on trunk muscle and core strengthening and stabilization training strategies suggests that core exercises contribute to enhanced athletic performance and likely reduce the risk of injury. In addition, from a programming standpoint, coaches can be flexible with their exercise prescription because: (1) core exercises are effective when performed in isolation (i.e., in separate training sessions) or combined with other training strategies (e.g., plyometric or strength-power exercises); and (2) shorter session duration (i.e., ≤ 30 min) may be superior for adaptations following core training, encouraging its application in time-constrained contexts.

## Leg Curl and Leg Extension

Leg curl and leg extension are the least frequently utilized exercises by Brazilian Olympic sprint and jump coaches (i.e., in conjunction with stiff-leg deadlift) (Table 2). These exercises are typically incorporated into resistance training routines as complementary exercises, for injury prevention, or during rehabilitation processes after muscle and joint injuries or surgical procedures (responses collected from open-ended questions; Loturco et al., 2023b). Considering that leg curl and leg extension are open chain exercises, it can be argued that their transfer to sport-specific movements (i.e., linear sprints and jumps) may be more limited compared to exercises executed in closed chain conditions (e.g., squats and deadlifts). This limitation stems from the weak relationships observed between jump and sprint performance and the muscle activation patterns in these exercises (Augustsson et al., 1998; Blackburn and Morrissey, 1998; Requena et al., 2009; Signorile et al., 1994). To some extent, this justifies the low frequency of use of these exercises by elite sprint and jump coaches in their training routines.

Another common application of leg curl and leg extension exercises is during isokinetic strength testing aimed at detecting muscle (or movement) imbalances and assessing injury risk (Deli et al., 2011; Housh et al., 1984; Yeung et al., 2009). To minimize these imbalances and mitigate the risk of injuries, these exercises are frequently incorporated into rehabilitation processes and preventive routines. For example, a 12-week intervention involving leg curl and leg extension exercises significantly reduced the knee pain scores of athletes from different sports disciplines (Cannell et al., 2001). Additionally, a resistance training program involving leg curl and leg extension exercises demonstrated effectiveness in reducinghamstring-to-quadriceps ratio imbalances after a 6-week intervention in well-trained soccer players (Śliwowski et al., 2015). Consequently, when the training goal is to assess and reduce muscular imbalances and facilitate a safe return to sports after an injury, these exercises are more commonly included in the resistance training routines of elite athletes. Moreover, the leg curl exercise is specifically implemented to increase hamstring strength, thereby reducing the injury risk associated with this muscle group (response collected from open-ended questions; Loturco et al., 2023b), a concern frequently reported by elite sprinters (Edouard et al., 2023). Indeed, several studies have integrated this exercise in hamstring injury prevention programs for athletes across various sports and training backgrounds (Bourne et al., 2018; Potier et al., 2009; Tous-Fajardo et al., 2006). Although leg curl and leg extension exercises are less frequently prescribed in the resistance training programs of elite athletes compared to more traditional and complex exercises (i.e., squats and Olympic weightlifting), they may be relevant in specific situations where athletes, under certain conditions, can benefit from their inclusion.

## Stiff-Leg Deadlift

The deadlift is a multi-joint exercise that can be divided into dynamic variations (e.g., a conventional or a sumo deadlift) or static-knee variations (e.g., a Romanian or a stiff-leg deadlift). In its dynamic variations, the knees perform flexion-extension, while in static-knee variations, the knees maintain a constant angle throughout the entire exercise (Coratella et al., 2022; Martín-Fuentes et al., 2020). Specifically, the stiff-leg deadlift (SLD) is performed with the knees extended throughout the entire range of motion (Coratella et al., 2022), indicating that the SLD is a predominantly hip-dominant variation exercise. This is a crucial aspect, as previous reports have highlighted the high electromyographic activity of the hamstring muscles during the SLD compared to other exercises (e.g., squats). For example, it was reported that the SLD involved the hamstrings to a higher degree than the back squat which exhibited only about half as much hamstring-integrated electromyography when compared to the SLD (Wright et al., 1999). Similarly, movements that emphasize hip extension, such as the SLD, appear to preferentially activate the biceps femoris long head, precisely the knee flexor muscle most commonly injured (i.e., affected or re-injured) (Marchiori et al., 2022; Opar et al., 2012). Consequently, it seems reasonable to consider the SLD as an important and valuable addition to any resistance training program, regardless of the sport discipline.

Curiously, only 5% of Brazilian Olympic sprint and jump coaches reported incorporating the SLD exercise into their resistance training routines (Loturco et al., 2023b). The low percentage of SLD usage is somewhat surprising, particularly considering the importance of hamstring muscles for sprinting performance (Zabaloy et al., 2023) and hamstring strain injury prevention programs (Bramah et al., 2023). For comparison purposes, a survey with Brazilian elite soccer strength and conditioning coaches revealed that Nordic curls, leg curls, and the SLD appear twice among the four most common types of exercises within the five main ranks of strength-power exercises prepared and prescribed by these coaches (Loturco et al., 2022a). In contrast, in amateur rugby (Zabaloy et al., 2022b), coaches reported not using either the SLD or any deadlift variations with their athletes. Overall, regardless of the sport, coaches and practitioners should consider the SLD as an important complimentary exercise. Instead of targeting a specific muscle region (e.g., the biceps femoris long head), it should be implemented as a pulling exercise variation to improve lower limb strength and power production, focused on promoting positive adaptations and increasing recruitment of posterior chain muscles (Coratella et al., 2022; Marchiori et al., 2022; Martín-Fuentes et al., 2020). In this sense, recent research demonstrated that the inclusion of the SLD in systematic resistance training programs induced increases in hamstring concentric strength and jump performance in rugby players (which can also benefit athletes from other sports) (Marchiori et al., 2022). Practitioners working in different areas and with different purposes (i.e., athletic performance or rehabilitation) may consider regularly incorporating this practical pulling exercise into their training schemes.

## Conclusions

Resistance training is unquestionably one of the primary and most commonly implemented strategies to enhance speed- and jump-related performance in numerous sports, regardless of age categories and training backgrounds. Undoubtedly, the methods and exercises used by coaches and practitioners vary according to their objectives, viewpoints, and most importantly, to the needs and demands of their athletes. In this extensive study, we present the resistance training practices adopted by coaches highly specialized in developing fast and powerful athletes (i.e., Olympic sprint and jump coaches). We firmly believe that this knowledge can assist practitioners from various sports in designing better and more sport-specific resistance training programs, particularly those aimed at optimizing sprint and jump qualities in elite athletes. The wide variety of topics and details provided here prevents us from objectively summarizing any general information concerning a specific resistance training method or exercise. On the other hand, the consistency and significance of these concepts and observations allow us to assert definitively: when well- programmed and prescribed, resistance training programs can be one of the most important (or perhaps the most important) allies in the development of more efficient and well-trained athletes. In addition, in essence, these precepts and principles can be applied to any sport, whether it is individual or collective (i.e., team-sports).
